# Test Bench for Evaluation of a Soft Robotic Link

**DOI:** 10.3389/frobt.2020.00027

**Published:** 2020-03-03

**Authors:** Lisbeth Mena, Concepción A. Monje, Luis Nagua, Jorge Muñoz, Carlos Balaguer

**Affiliations:** Robotics Lab, Carlos III University of Madrid, Madrid, Spain

**Keywords:** soft robotics, soft link, test bench platform, kinematics model, sensorial system, control system

## Abstract

In this paper we describe the control approaches tested in the improved version of an existing soft robotic neck with two Degrees Of Freedom (DOF), able to achieve flexion, extension, and lateral bending movements similar to those of a human neck. The design is based on a cable-driven mechanism consisting of a spring acting as a cervical spine and three servomotor actuated tendons that let the neck to reach all desired postures. The prototype was manufactured using a 3D printer. Two control approaches are proposed and tested experimentally: a motor position approach using encoder feedback and a tip position approach using Inertial Measurement Unit (IMU) feedback, both applying fractional-order controllers. The platform operation is tested for different load configurations so that the robustness of the system can be checked.

## 1. Introduction

Soft robotics is a new field of interest in the robotic community, which is growing quickly. The most important contributions research are from institutions in the United States, Europe, and Asia as seen in Bao et al. (2018). The use of soft materials and variable stiffness technologies in robotics represents a new generation of machines (Laschi et al., 2016) and also new challenges for researchers in the field, such as the development of complex structures and actuators, and the sensing, modeling, control, and manufacturing techniques for soft materials.

The first soft robots were inspired by muscular hydrostats, tentacles of cephalopods, and the trunks of elephants. These were used for manipulation, such as the OctArm, presented in Grissom et al. (2006). The OctArm is pneumatically actuated, and it has a backbone without joints. Even if pneumatic actuation has long been used in robotics, a new class of soft actuators known as Pneu-Net (pneumatic networks) are emerging. This class of soft actuators originally developed by the Whitesides Research Group at Harvard is widely used in soft robotics (Laschi et al., 2016). For instance, a Pneu-Net actuated robot totally made with soft materials (elastomeric polymers) featuring quadrupedal locomotion is presented in Shepherd et al. (2011).

Shape Memory Alloy (SMA) actuator technology is also extensively used in soft robots such as: GoQBot by Lin et al. (2011), OCTOPUS robot developed by Laschi et al. (2012), or Meshworm presented in Seok et al. (2012).

Soft robots have been developed to be able to perform different skills. For example, Stickybot in Kim et al. (2008) is a climbing robot, PLANTOID in Sadeghi et al. (2014) is able to grow in the same way that a plant root does. PoseiDRONE in Calisti et al. (2015) is a robot capable of underwater legged locomotion.

Soft robots can also adapt to variable environments, perform adapting to the task requirements or physical constraints, and manipulate unknown objects that vary in size and shape. Their morphology can be adapted to the environment, as bioinspired robots. The advantages of soft robots are about simplicity of design, accessibility, and adaptability to better interact with natural, unstructured environments and with humans (Laschi et al., 2016).

Soft materials are less dense than stiff ones, so the robots can be made much lighter and require smaller actuators. Also, these materials make the robot more resilient and able to withstand impacts from falls. These materials will absorb vibrations from contact with the environment. Soft robots are being built to operate in human environments, so it is inevitable that they will become more and more like us (Trimmer, 2015).

However, the challenges are important, since the conventional robotic techniques are not applicable. Soft robotics requires a combination of advanced fabrication techniques to include rigid and soft structures. There are also obstacles in the way of commercialization. In Bao et al. (2018), the authors propose the development of a common fundamental theory for robot design and control for soft robotics to be an independent discipline.

Humanoid robots are usually built from metal and plastic components. These robots are generally quite stiff and do not make extensive use of soft materials in their joints or contact surfaces (Trimmer, 2015), but soft robotics is slowly being introduced, usually based on human body biomechanics and locomotion. For example, compliant and elastic elements to produce soft motions have been used in Stoelen et al. (2016) to build a compliant robot arm.

The introduction of soft robotics in humanoid robots is still partial, that is, some parts are being replaced in classic humanoids, maybe to ensemble all of them in a complete soft humanoid in the future.

Manipulation and grasping are the most popular abilities developed in soft humanoids. For example, RBO Hand 2 is a pneumatically actuated hand presented in Deimel and Brock (2016). In Feng et al. (2018), a soft robotic hand is introduced, where each single finger has a three-stage cavity structure. Another soft robotic hand is BCL-13, presented in Zhou et al. (2018). It has 13 Degrees of Freedom (DOF) with four fingers and solenoid valve actuators, and represents an intuitive grasping control approach. The soft manipulator SIMBA developed by Mishra et al. (2017) proposes a soft re-configurable hand, that adapts the fingers according to the shape and size of the object.

In addition, other components for soft humanoid robots are being developed: a flexible spine structure and tendon(muscle)-driven system, developed by Mizuuchi et al. (2002); a robotic eyeball inspired by nature that employs a soft actuator combining three linear dielectric elastomer actuators (Li et al., 2018); a minimalistic model of leg structure with biarticular tension spring (Iida et al., 2008); and an structurally flexible humanoid spine based on a tendon-driven elastic continuum acting as a neck (Reinecke et al., 2016).

In the case of mechanisms that simulate human necks on robots, two configurations stand out: series and parallel. The serial neck configuration is often used for its control simplicity, since each DOF is commanded independently, as in HRP-4 (Hirukawa et al., 2004) and Asimo-2002 Honda (Sakagami et al., 2002). However, parallel mechanisms are closer to a human neck configuration. This type of mechanism is interesting because it reduces the number of actuators and sensors for closed-loop control as shown in Nori et al. (2007), where the neck is surrounded by steel tendons in place of muscles. For the iCub robot (Beira et al., 2006) two parallel mechanisms were implemented for the neck. The first one is based on a spring with three actuated cables; the second one uses a three DOF parallel mechanism with a central passive spherical strut.

Research has been performed in order to propose a new soft robotic neck design different from the rigid ones seen before. The authors in Reinecke et al. (2016) present an anthropomorphic neck prototype with three DOF using a continuum mechanism based on tendons for actuation, where the structure of the neck is silicone. Another neck proposal is presented in Gao et al. (2012), where a compression spring is used in two DOF and four cables are used to actuate the mechanism in an antagonistic fashion. The authors in Beira et al. (2006) introduce the iCub head describing the cable-driven mechanism based on a spring link.

A common aspect in the majority of the works cited above is that the control of the soft neck is not addressed, and a study of the robustness of the system to head load variations is missing. Our research focuses on this specific topic. It is based on the soft neck described in Nagua et al. (2018), where a parallel mechanism configuration mimics the structure of the human neck. The motors and cables act as muscles and tendons, and a central soft link acts as cervical spine, allowing movements of flexion, extension and lateral bending of the end-effector (tip). The design proposed is compact and portable, and could be easily embedded into any humanoid's torso.

Due to its design characteristics, the gain of the system modeling the neck is different depending on the neck position, making the platform specially suitable as a test bench for robust controllers. For instance, in Nagua et al. (2018) a low level robust control based on fractional order controllers is proposed and tested. In addition, different payloads can be applied at the tip allowing performance comparison and testing under different load configurations.

The use of fractional calculus in robust control allows a wider range of solutions. This makes controllers to better shape the system, usually taking Bode's ideal transfer function (Bode, 1945) as a reference. Some examples of fractional order controllers can be found in Petras (2009), Monje et al. (2008a), Qingshun et al. (2015), and Ranjbaran and Tabatabaei (2018), to cite a few. A detailed comparative can be found in Xue and Chen (2002).

Podlubny's(1999) Fractional Proportional Integral Derivative controller (PI^*ei*^D^*ed*^, where *ed* and *ei* are the fractional derivative and integral exponents) is often used, but in this work, as the control specification can be reached using a simpler controller, just the PI^*ei*^ case will be considered.

The main problem found in our previous work (Nagua et al., 2018) is the kinematic mismatch between the theoretical and observed tip angles. That is, although the correct motor positions can be reached according to encoders, there is a remarkable difference between the expected and the real neck angles, mostly when different payloads are applied at the tip.

As this kinematic mismatch is a major issue, the aim of this work is to improve the soft neck performance through the introduction of a low cost IMU sensor, that will allow the direct measurement of the tilt and orientation angles of the neck. Besides, a control loop will be closed with the IMU data in order to guarantee a robust performance of the neck to load variations, using a fractional order controller.

## 2. Platform description

**Figure 1:**
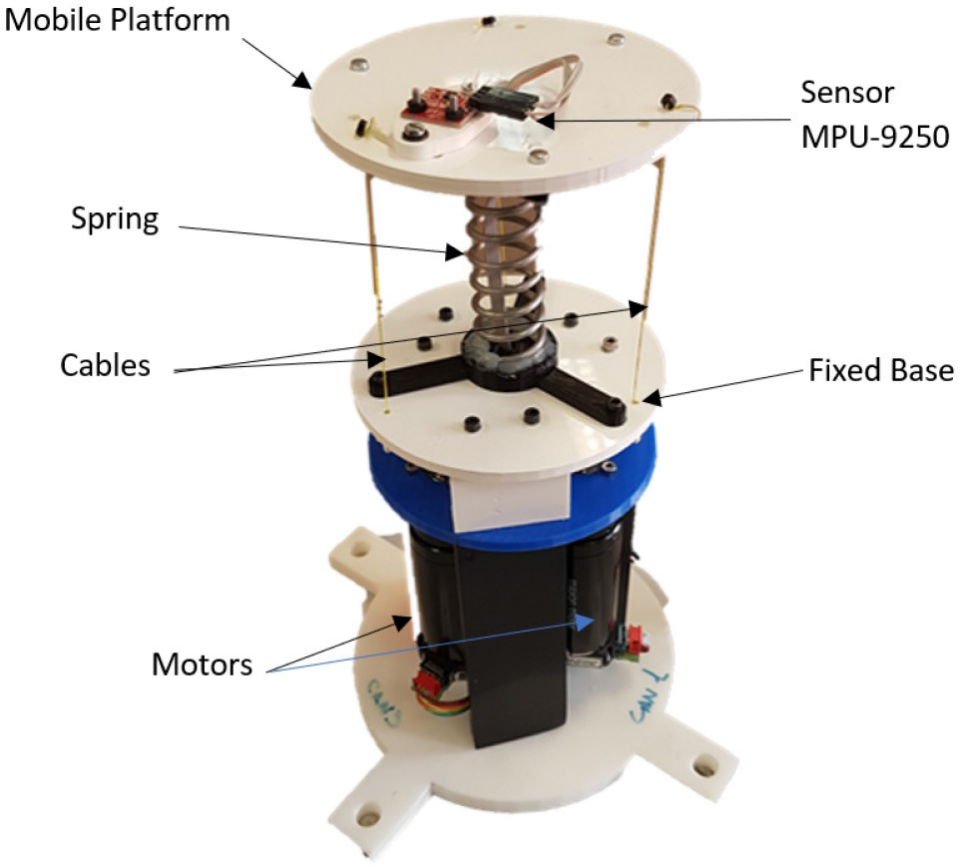
Soft neck platform.

Shown in Figure 1, the neck is made up of a central mechanical soft link, which acts as the backbone, and a parallel mechanism driven by cables, which produces the flexion of the central mechanical soft link. In this way the platform can reach any tilt and orientation taking into consideration the operating limits established by the maximum permissible flexion and the route of the cables.

The parts of the neck are:

1. Base
2. Mobile platform
3. Mechanical soft link
4. Tendons
5. Motors.

In order to build a prototype, an initial design was made and validated in a CAD software, through which the schematics of the components that constitute the platform were obtained. These components were then manufactured in a 3D printer and assembled making a neck prototype. Furthermore, a position control loop was implemented using the platform motor encoders as sensors. This control loop was designed following the same scheme and specifications as in Nagua et al. (2018).

All parts of the prototype can be built using 3D printing, except for the mechanical soft link (spring). In this case, the link has a weight of 100 g for a payload of 600 g, which means a ratio of 600% load to weight of the link (excluding all other parts of the prototype).

The cables or tendons are driven by means of three independent actuators located in the base, composed by a set of driver, motor, and gear with the following characteristics:

- Driver: Technosoft iPOS4808 MX-CAN; 400 W, 12–50 Volt, 8 Amp (intelligent motor driver)
- Motor: Maxon RE 35; graphite brushes, 42 Volt, 90 Watt
- Gear: Maxon planetary gearhead GP32A (3.7 : 1).

The different positions that the robot can reach are defined by tilt and orientation parameters as shown in Figure 1. After defining a desired position, it is necessary to solve the inverse kinematics as described in Nagua et al. (2018) to obtain the proper lengths for the cables, and thus calculate the angular positions required in the actuators that will allow the platform to reach a desired position in orientation and tilt.

## 3. Platform Kinematics

**Figure 2:**
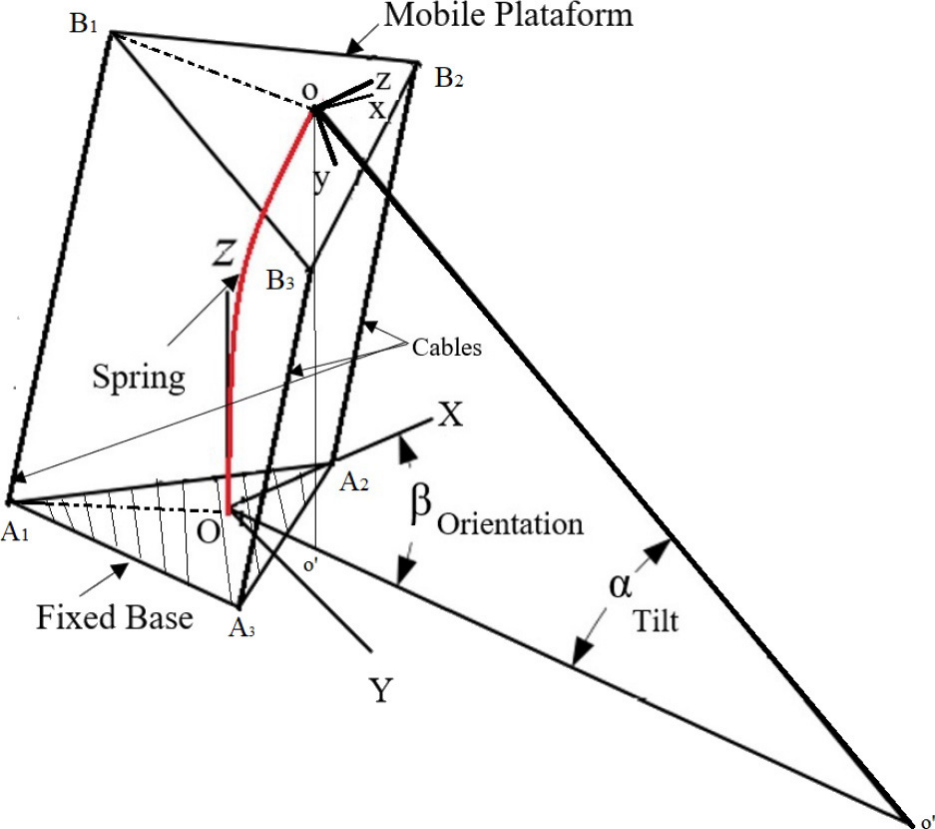
Schematic diagram of the soft robotic neck. Coordinate frame OXYZ refers to the fixed base and frame oxyz is attached to the moving platform.

The soft neck shown in Figure 1 have two independent parameters: angle α (tilt angle) and angle β (orientation angle). These parameters represent the position we want to obtain of the robotic neck as shown in Figure 2. The inverse kinematics problem is now how to get the lengths of the cables that allow this positioning.

Equation (1) represents the lengths of cables needed to obtain a given position (Nagua et al., 2018),

(1) Li=‖To′OoBi→−OAi→‖

where:

- TOo′ is the homogeneous transformation matrix that represents the projection from *oxyz* (frame mobile) to *OXYZ* (frame fixed):
  (2) TOo′=[ROo′Po01]
  where *P*_*o*_ is the position vector of point *o* with respect to the base coordinate frame and ROo′ is the rotational matrix that describes the orientation of the moving platform using the Euler angles with orientation *ZYZ*.
- ${\vec{oB}}_{i}$ represents the points of the mobile platform.
- ${\vec{OA}}_{i}$ represents the points of the fixed base.

To solve the inverse kinematics we must consider the lateral bending curve of the spring spine considering β_*s*_ = β_0_*L*/*L*_0_ as the flexural rigidity after compression of the spring (Timoshenko, 1936). Additionally, the inertia *I* and bending constant β_*o*_ of the spring must be calculated with the following equations:

(3) $$\begin{array}{l} I=\frac{\pi d^{4}}{64};\;\beta_{o}=\frac{2EGIL_{0}}{\pi N_{a}\frac{d}{2}\left(E+2G\right)} \end{array}$$

The spring is made of A228 steel and Table 1 presents its characteristics.

**Table 1:** Parameters of the selected compressive spring.

**Parameter**	**Value**	**Unit**
D: diameter of the spring (helix)	0,03	[m]
*L*_*o*_: length of spring	0.1	[m]
d: diameter of the wire	0.0025	[m]
*N*_*a*_ is the number of coils	12	
G: is the shearing modulus	80	[GPa]
E: the elastic modulus	200	[GPa]

The problem of direct kinematics is to calculate the angles of orientation β and tilt α knowing the robotic neck posture, that is, knowing the lengths of the cables $L={\left[\begin{array}{ccc} L_{1}, & L_{2}, & L_{3} \end{array}\right]}^{T}$. This length data are obtained from the DC motor enconders.

We use Matlab function fsolver to solve the direct kinematics based on Equation (1).

## 4. Control Problem

**Figure 3:**
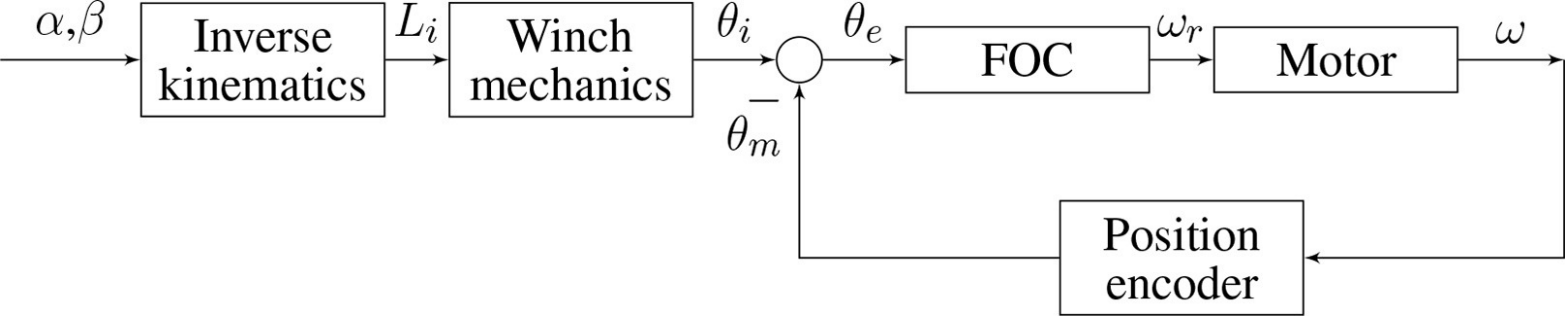
Soft neck control scheme for a single motor, showing: Angular position error (θ_*e*_), Fractional Order Controller (FOC), Motor, Angular speed (ω), Position encoder, and Angular position (θ_*m*_).

In order to reach and hold the desired motor positions (θ_*m*_), the position feedback control described in Figure 3 will be used, where position error is obtained from encoder data and computed through a fractional order controller, resulting in a velocity control action.

In this control scheme, the motor block input is modeled as velocity command and the output is modeled as position. Therefore, the system model is composed of a first order transfer function modeling velocity plus an integrator and a gain modeling the encoder sensor. At the same time, the motor internal model is known, consisting of a velocity feedback loop, featuring a fractional proportional integral controller (foPI). Due to the complexity of the internal loop, a simplified model obtained from the experimental identification of the motors was found, which results in the following equation:

(4) $$\begin{array}{l} G\left(s\right)=\frac{54.89}{54.89+s}\cdot \frac{k_{enc}}{s}, \end{array}$$

where *k*_*enc*_ = 6 is the encoder gain that converts *rpm* input into *deg*/*s* (360/60).

**Figure 4:**
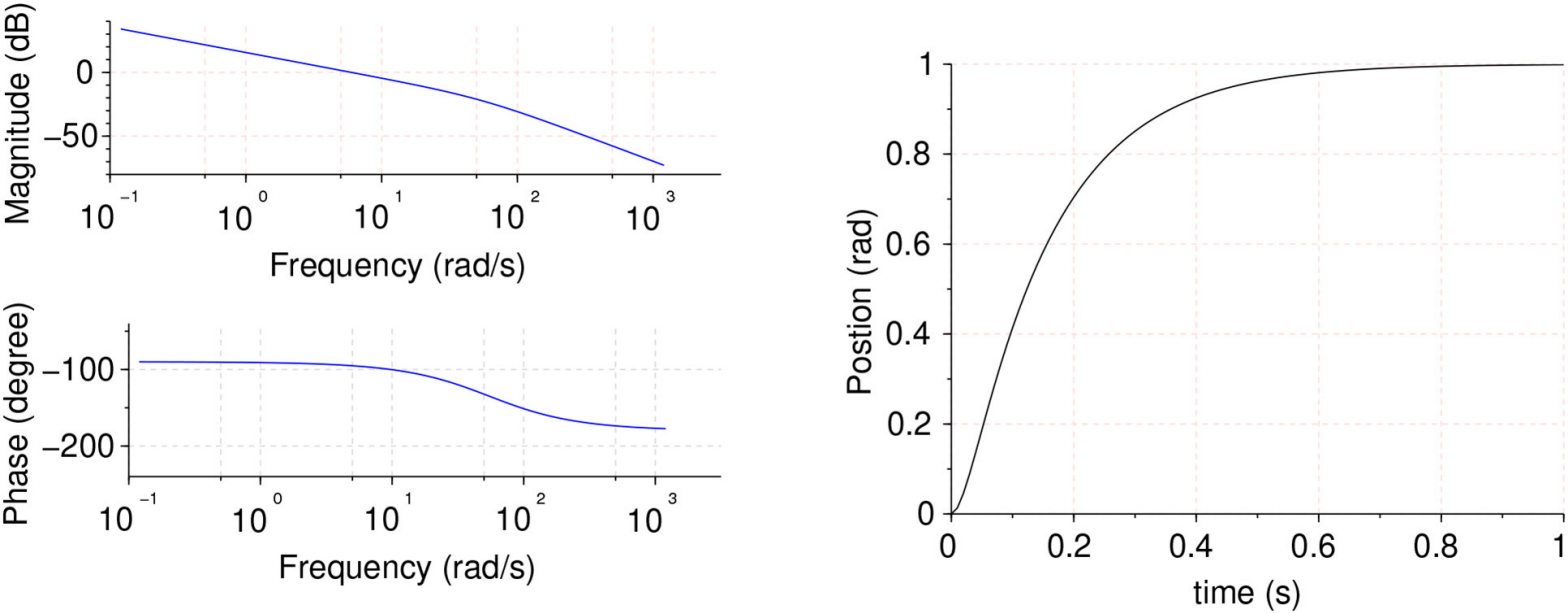
Motor model frequency response **(Left)** and time unitary feedback response **(Right)**.

Motor model time and frequency responses are shown in Figure 4.

The fractional controller will be tuned using the method described in Monje et al. (2008b), with a flat phase slope specification, ensuring a robust control in order to deal with model uncertainties and mass changes. Crossover frequency and phase margin specifications were chosen as follows:

- ω_*cg*_ = 12*rad*/*s*
- ϕ_*m*_ = 60*deg*

**Figure 5:**
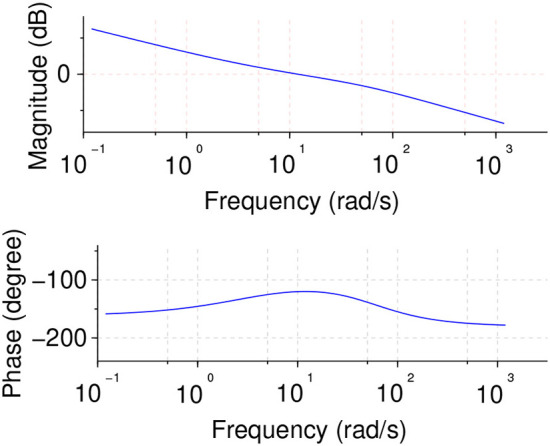
Bode diagram for cascaded system and controller in open loop showing the flat phase slope specification.

The controller parameters obtained using Monje's method are *kp*:1.7807044; *ki*:4.8890371; *ei*:−0.81, resulting in the following controller transfer function:

(5) $$\begin{array}{l} C\left(s\right)=1.7595866+4.7817906s^{-0.8}, \end{array}$$

and the Bode diagram for the system and controller in open loop configuration is shown in Figure 5.

Although motor positions will be achieved efficiently with a robust performance thanks to the control strategy, the problem of the kinematics mismatch still needs a solution. In order to measure that problem, the actual neck inclination and bending angles will be obtained from sensors.

## 5. Soft Neck Sensory System

The soft neck prototype estimates its final position using the kinematics through motors encoders signals. However, there is a visual difference between the expected and real neck position when loads are applied. The contribution of this work is to improve the platform including an IMU sensor to obtain real data on the neck angles, which will allow to close an external position control loop. The sensor selected is the low cost MPU-9250, which is a 9-axis Motion Tracking device that combines 3-axis gyroscope, 3-axis accelerometer, 3-axis magnetometer, and a Digital Motion Processor™ (DMP), all in a small 3 x 3 x 1 mm package. This sensor was selected for its high reliability and because it meets the necessary speed specifications required for measuring tilt and orientation angles in this platform.

### 5.1. Library

The *I*^2^*C* communication MPU-9250 sensor allows direct connection and communication with Arduino development boards, which will be used as data acquisition card in this case. Sparkfun markets the MPU-9250 sensor and they have developed an open source library in C++, compatible with the Arduino IDE. It consists of two libraries:

- Library MPU9250: Allows to obtain the data of the accelerometer, gyroscope, and magnetometer in raw format.
- Library quaternionFilters: Calculates the corresponding sensor angles yaw, pitch and roll, through each of the components obtained by MPU-9250.

The quaterniosFilters library implements an orientation filter for matrices of inertial and magnetic sensors as proposed in Madgwick (2010), where acceleration, rotation speed, and magnetic moments are merged to produce an estimate based on quaternios of absolute orientation of the device, which can be converted into roll, pitch and yaw angles. The performance of the filter is as good as conventional Kalman-based filters, with lower computational cost.

In this case, the Mahony filter has been used, which uses a similar scheme to Madgwick but it employs a proportional and integral filtering in the error between the estimated and measured reference vectors (Mahony et al., 2005).

### 5.2. Initial Calibration

The IMU calibration is carried out before mounting the sensor in the mobile platform in order to provide wider movements during the calibration process and avoid the limitations of the neck working space. For the initial data acquisition the magnetometer must be calibrated in order to decrease the reading errors. For this a function is used, which accumulates magnetometer data after device initialization. It calculates the bias and scale in *X*, *Y*, and *Z* axes. It waits 4 s to get ready followed by 15 s of magnetometer data sampling. And all this is done while moving the sensor following a trajectory that shapes number eight or the infinity symbol. As a result, the sensor is calibrated with the following magnetometer offset values in each axes: *MagX* = 49.53, *MagY* = 633.23, and *MagZ* = 451.66 [*G*]. Finally, the IMU sensor is mounted in the upper plane of the mobile platform, as illustrated in Figure 1.

Once the sensor has been embedded, a compensation strategy is used to obtain the real pitch and roll measurements. For this purpose, the pitch and roll angle data of the sensor are processed, calculating the recursive average of the first 500 data read of each angle using Equations (6, 7). The resulting value is considered as an initial offset for pitch and roll angles, respectively. The calculation of the final inclination and orientation is made from the reading of the sensor angles, subtracting the respective offset values (Equations 8, 9) in order to ensure a relative initialization of the sensor, considering the mobile platform initial position. In a future work we will use a Motion Capture (MOCAP) system in order to validate the IMU measurements and correct the absolute error committed, if necessary.

(6) $$\begin{array}{l} \theta_{offset}=\frac{\bar{\theta }}{i}\left(i-1\right)+\frac{\theta_{IMU}}{i} \end{array}$$

(7) $$\begin{array}{l} \phi_{offset}=\frac{\bar{\phi }}{i}\left(i-1\right)+\frac{\phi_{IMU}}{i} \end{array}$$

(8) $$\begin{array}{l} pitch=\theta_{IMU}-\theta_{offset} \end{array}$$

(9) $$\begin{array}{l} roll=\phi_{IMU}-\phi_{offset} \end{array}$$

Now, pitch and roll offset values are the resource for calculating the tilt and orientation angles of the soft neck platform, according to subsection 5.3.

### 5.3. Conversion of Angles

The sensor library establishes an axes and rotation configuration as: the accelerometer axis *X* is aligned with the axis *Y* of the magnetometer, so the axis *Z* of the magnetometer is opposite in the direction of the axis *Z* of the accelerometer and the gyroscope.

**Figure 6:**
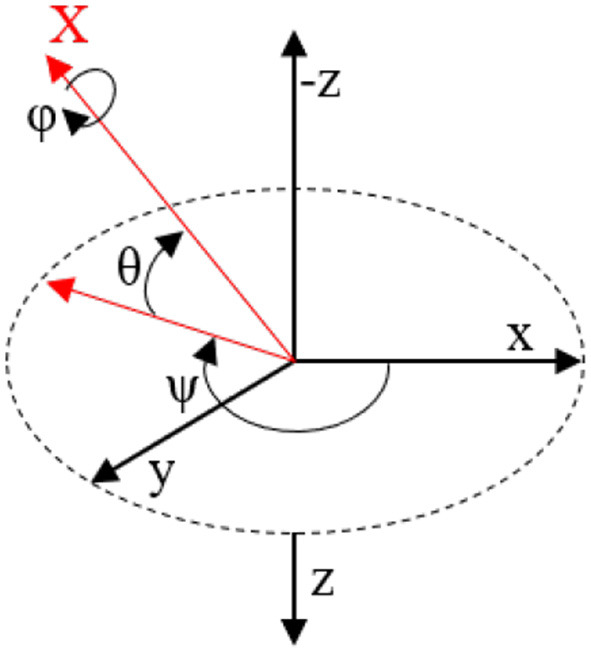
Tait-Bryan angles, yaw (ψ), pitch (θ), and y roll (ϕ).

The library generates the quaternium from which the angles yaw, pitch, and roll are obtained. These are the Tait-Bryan angles, commonly used in aircraft orientation (see Figure 6).

In this coordinate system, the positive *Z* axis is down to the ground. Yaw is the angle between the sensor's *X* axis and the Earth's magnetic north. Pitch is the angle between the *X* axis of the sensor and the earth plane; toward earth it is positive and toward sky it is negative. Roll is the angle between the sensor *Y* axis and the earth plane; the upward *Y* axis is positive roll.

These angles come from the definition of the homogeneous rotation matrix constructed by quaternions. Tait-Bryan angles, as well as Euler angles, are not commutative; that is, to get the correct orientation, the rotations must be applied in the correct order, which for this configuration is yaw, pitch and roll.

The Tait-Bryan matrix with the corresponding angles yaw (ψ), pitch (θ), and roll (ϕ) looks as follows:

(10) $$\begin{array}{l} R_{\phi ,\psi ,\theta }=\left[\begin{array}{ccc} C\psi C\theta & \left(C\psi S\theta S\phi -S\psi C\phi \right) & \left(C\psi S\theta C\phi +S\psi S\phi \right)\\ S\psi C\theta & \left(S\psi S\theta S\phi +C\psi C\phi \right) & \left(S\psi S\theta C\phi -C\psi S\phi \right)\\ -S\theta & C\theta S\phi & C\theta C\phi \end{array}\right] \end{array}$$

Only *R*_θ_ is considered because angle ψ corresponding to the angle *Yaw* is annulled for the neck platform, since it only has two DOF: one for tilt in *Pitch* and the other for orientation in *Roll*. So the Tait-Bryan matrix is reduced to the vector:

(11) $$\begin{array}{l} R_{TB}=\left[\begin{array}{c} S\theta C\phi \\ -S\phi \\ C\theta C\phi \end{array}\right] \end{array}$$

The tilt angle (α) on the platform is the projection of the vector *R*_*TB*_ on the *Z* axis, according to the following equations:

(12) $$\begin{array}{l} cos\alpha =\frac{R_{TB}.Z}{\left||R_{TB}||.||Z|\right|}=\frac{\left[\begin{array}{c} S\theta C\phi \\ -S\phi \\ C\theta C\phi \end{array}\right]\;.\;\left[\begin{array}{c} 0\\ 0\\ 1 \end{array}\right]}{\left||R_{TB}||.||Z|\right|} \end{array}$$

(13) $$\begin{array}{l} cos\alpha =\frac{C\theta C\phi }{\sqrt{{\left(S\theta C\phi \right)}^{2}+{\left(-S\phi \right)}^{2}+{\left(C\theta C\phi \right)}^{2}}} \end{array}$$

The orientation angle (β) is obtained from the projection of the vector *R*_*TB*_ on the *XY* plane. In other words, there are two possible complementary resolutions for orientation. In this case, we consider the solution of the *X* axis that does not need to add arithmetic calculations for the absolute angle.

(14) $$\begin{array}{l} cos\beta =\frac{R_{TB}.Z}{\left||R_{TB}||.||Z|\right|}=\frac{\left[\begin{array}{c} S\theta C\phi \\ -S\phi \\ C\theta C\phi \end{array}\right]\;.\;\left[\begin{array}{c} 0\\ 0\\ 1 \end{array}\right]}{\left||R_{TB}||.||Z|\right|} \end{array}$$

(15) $$\begin{array}{l} cos\beta =\frac{S\theta C\phi }{\sqrt{{\left(S\theta C\phi \right)}^{2}+{\left(-S\phi \right)}^{2}}} \end{array}$$

### 5.4. Communication

The MPU-9250 sensor is managed by Arduino development board via *I*^2^*C* communication, connected to the SCL, SDA, VDD, and GND pins. The SCL pin is the *I*^2^*C* serial clock with 100 or 400 kHz frequency, and the SDA pin transfers the *I*^2^*C* serial data. The soft neck platform is controlled via CAN bus and C++ using Qt creator IDE, from Ubuntu.

The connection between Arduino card and Qt was made by serial communication, with a speed of 9600 bauds. A C++ library was generated to connect computer and Arduino, with the following configurations: enabling a serial COM port, port search with Arduino identifiers and sensor data acquisition.

The tilt and orientation sensor data is obtained reading and writing in the serial port with the following algorithm:

1. Writing from Qt of a request character in the port.
2. Reading from Arduino of the request character.
3. Sending the sensor data from Arduino by writing an identifying character of the tilt data and another identifying character of the orientation data separated by a comma (,) (*i tilt data, o orientation data*).
4. Qt waits and reads the port with data sent by Arduino.
5. Qt considers the data identification characters and the comma for separating, sorting, and recognizing the tilt and orientation data.

With the sensor data obtained, the compensation strategy presented in section 5.2 is used in order extract the real pitch and roll angles.

## 6. Tests and results

Once the operation of the sensor has been checked, an experimental test was carried out on the platform to validate the system. This test consisted on moving the neck two rounds of 360*deg* with steps of 10*deg* in orientation, and 15*deg* for the first round and 25*deg* for the second round in tilt angle. Time between steps was 1 s. Additionally, load tests were performed with 0, 500, and 1,000 gr placed on the top platform. The controller used in each motor is an integral fractional order controller with ω_*cg*_ = 12*rad*/*s*, ϕ_*m*_ = 60*deg* and parameters *kp*:1.7807044; *ki*:4.8890371; *ei*:−0.81.

**Figure 7:**
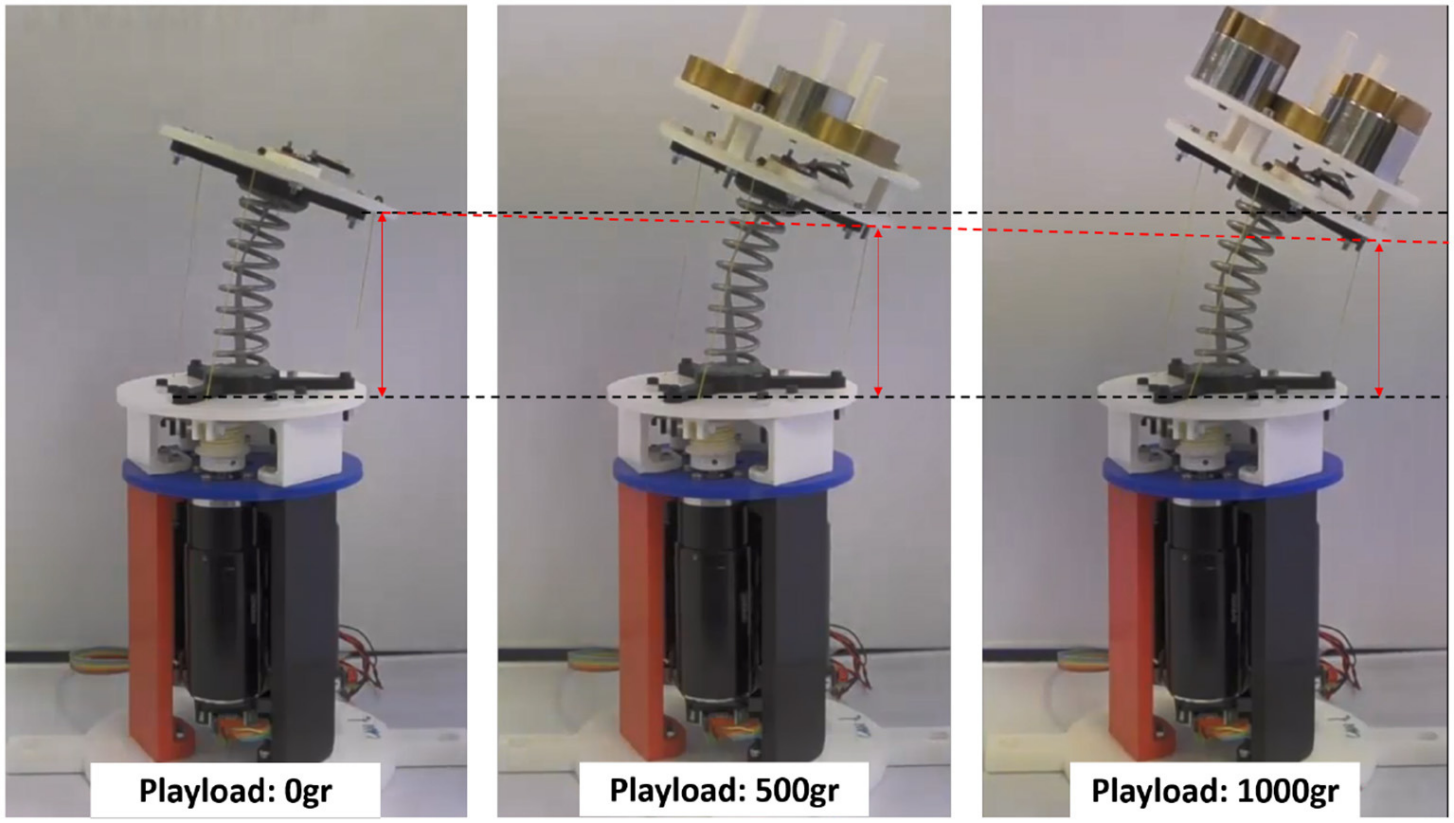
Experiment test with different playload.

The tests can be visualized in the video http://cort.as/-JY98, where the neck operation with different loads is shown. As seen in the video and in Figure 7, the actual tilt of the platform is slightly different for each load, even though the controllers act appropriately maintaining the kinematics calculated values, mostly when tilt target is set on 25*deg*.

**Figure 8:**
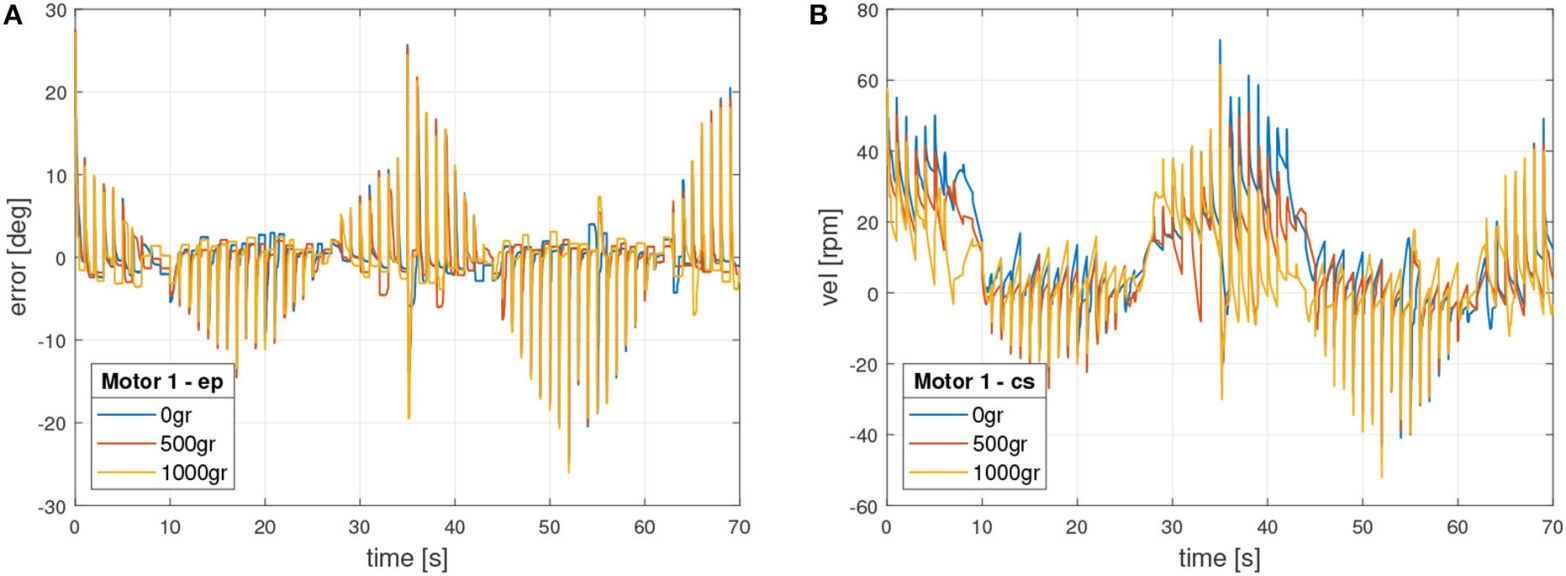
Motor 1 encoder signal (θ_*m*_) vs. kinematics, with different playloads.

The position encoder data allows verifying both kinematics and control calculation. Figure 8 shows a comparative graph between kinematics estimated position and motor 1 encoder signal for each load test.

**Figure 9:**
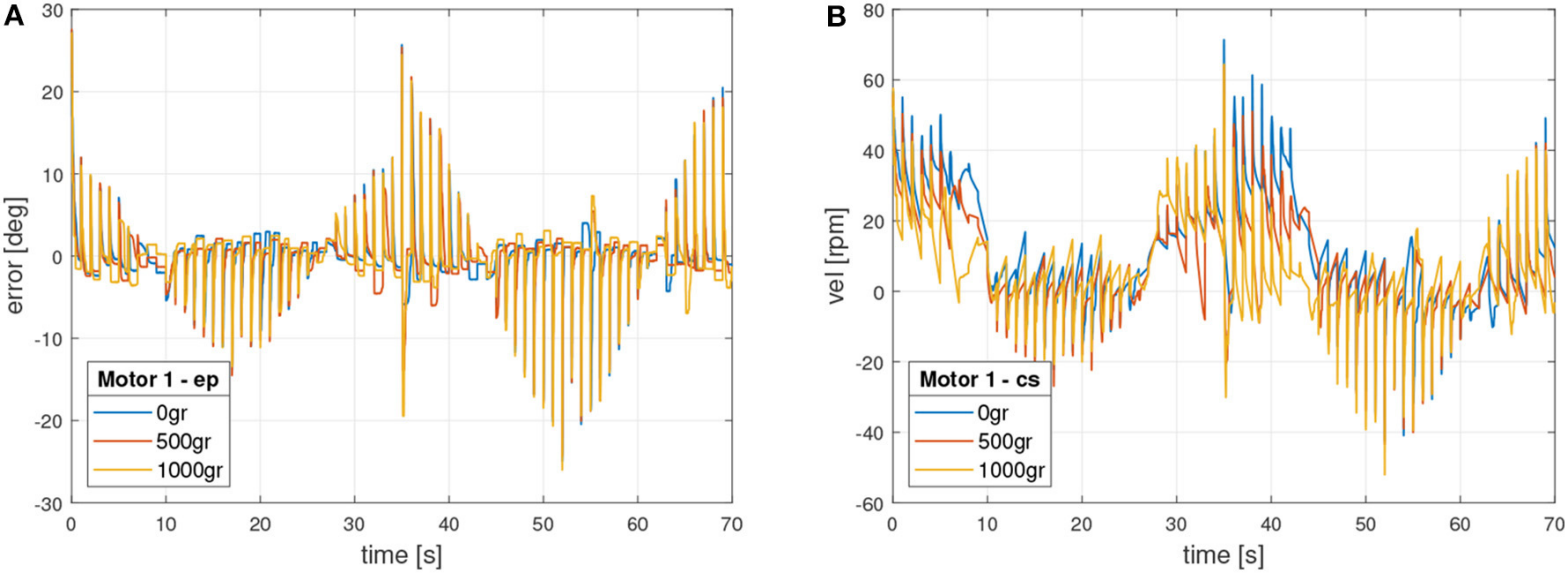
Motor 1 external control signals. **(A)** Position error signal. **(B)** Velocity control signal (*u*).

Additionally, control signals were validated in accordance with section 4. Figure 9 shows the external control signals in motor 1, which include position error signal and control signal in velocity.

**Figure 10:**
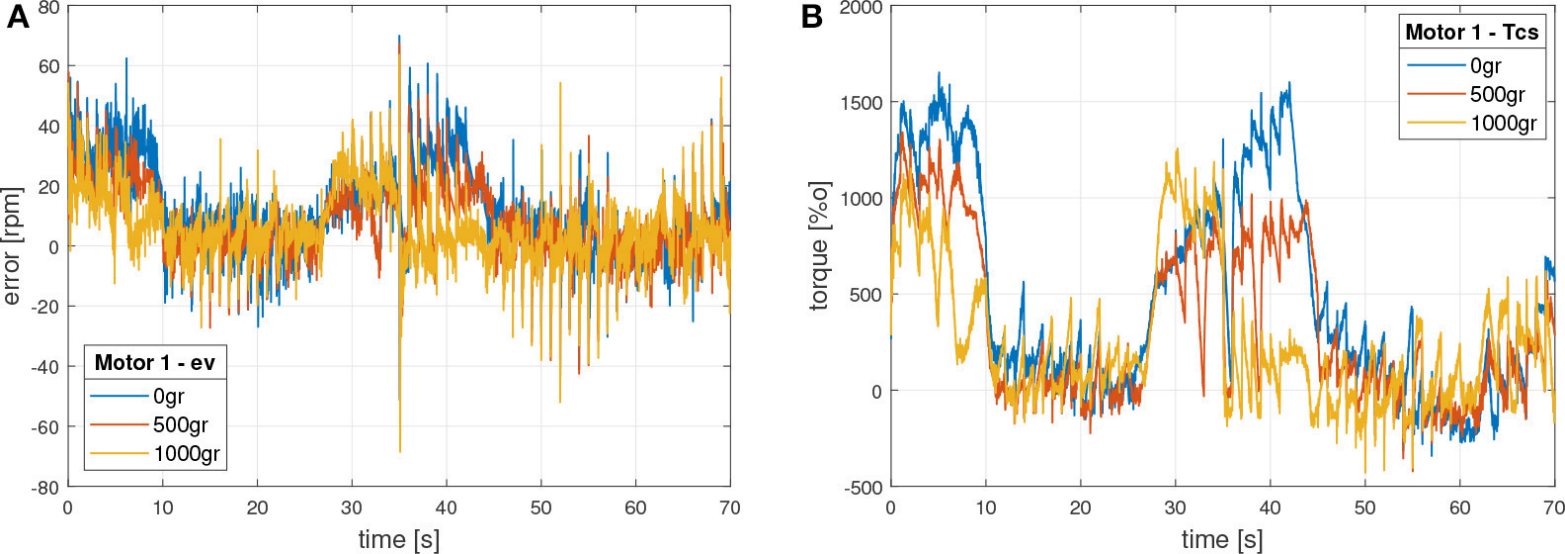
Motor 1 internal control signals. **(A)** Velocity error signal. **(B)** Torque control signal.

On the other side, Figure 10 presents the internal control signals in motor 1. In this case the error is in velocity and the control signal in torque, which commands directly the motor.

**Figure 11:**
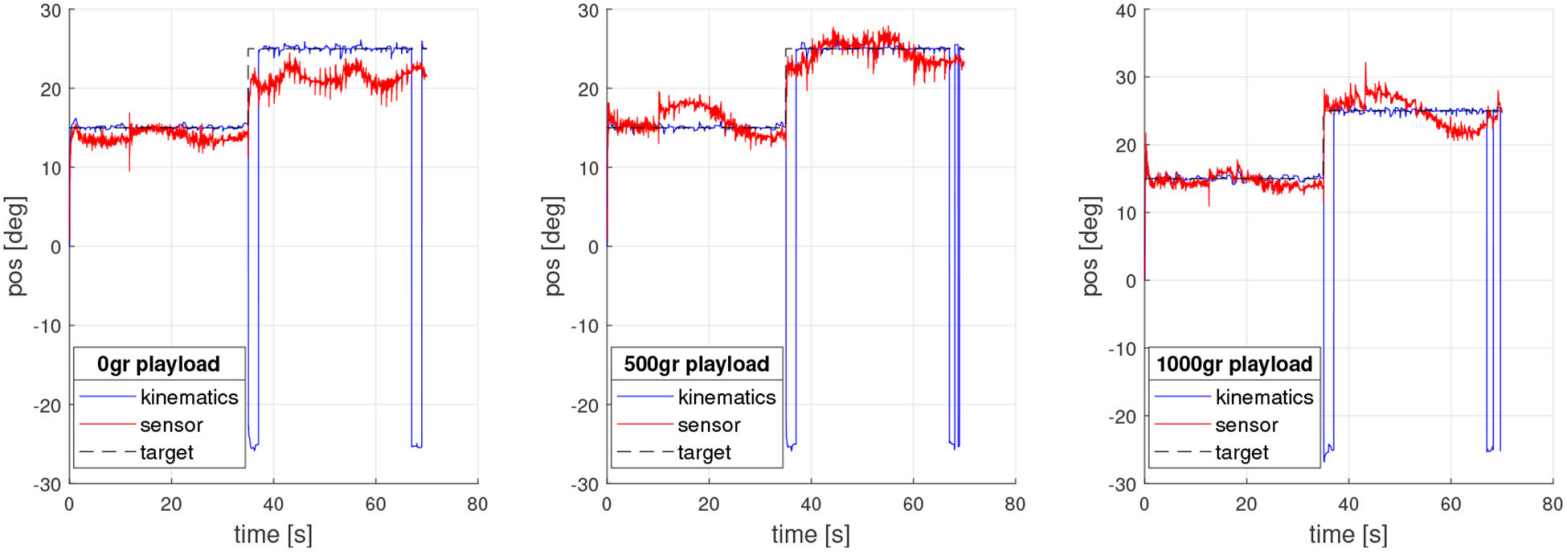
Tilt sensor response for experiment with different playloads.

**Figure 12:**
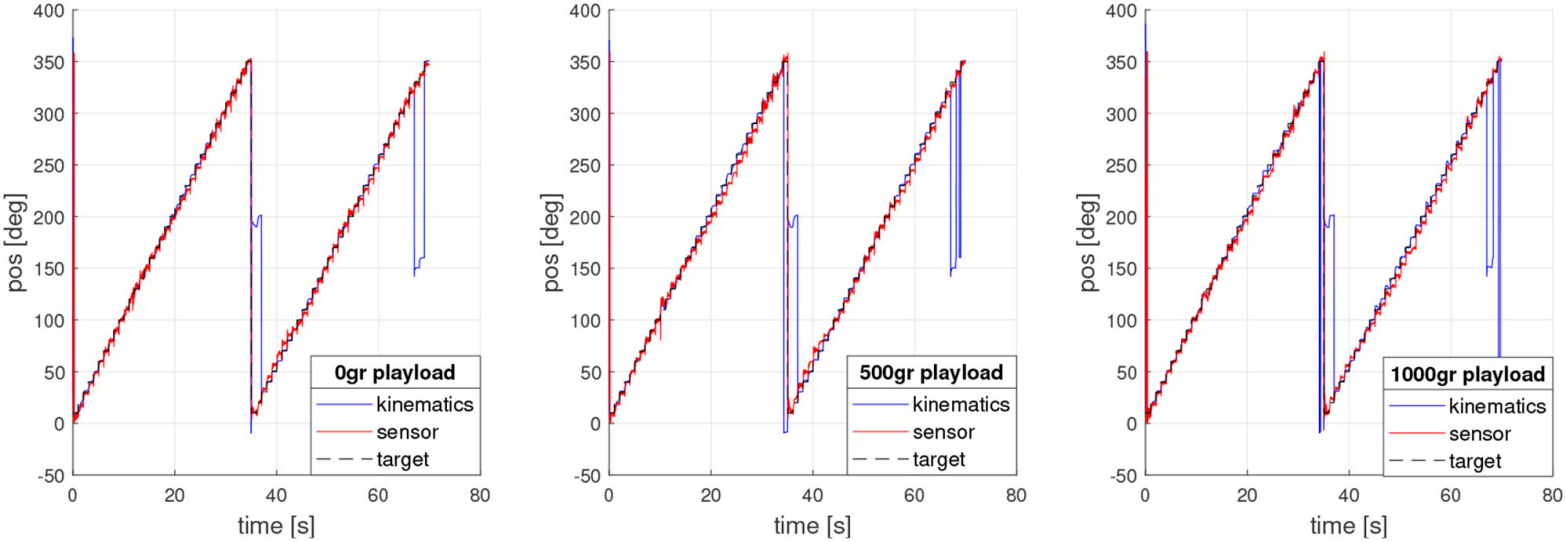
Orientation sensor response for experiment with different playload.

Once the test was performed, it was possible to verify the kinematics, as shown in Figures 11, 12, both for tilt and orientation angles, respectively. The kinematics is concurrent with the fixed target, which was computed using data obtained from the encoders of each motor (1, 2, and 3)and estimating the tilt and orientation angles according to the kinematic model reviewed in section 3.

However, the data obtained from the sensor prove that, at higher loads, the platform physically exceeds the value of the tilt target. In addition, it is possible to observe the oscillations in the movement produced by the flexible link composed of a spring. In the future a soft material neck will be implemented. Additionally, this behavior could be produced by the strong simplification of the kinematic model.

Therefore, the implementation of the inertial sensor in the platform is an important improvement and it will allow to close a new control loop in order to improve the behavior of the neck.

## 7. Closed loop inclination control

Once verified the mismatches between theoretical results and actual platform inclinations, a closed loop control is proposed in order to avoid the steady state inclination error. As shown in section 6, inclination values show important mismatches that have to be canceled through a feedback control loop. This can be achieved through the inclination error measured with the IMU sensor and an fractional integral controller scheme.

**Figure 13:**
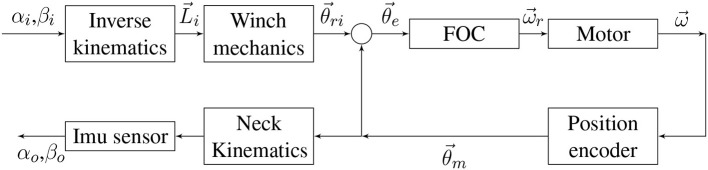
Soft neck system (Plant), showing input (α_*i*_,β_*i*_) and output (α_*o*_,β_*o*_).

The new control variables are tip orientation and inclination; therefore, the new system comprises the entire neck (including spring and motors) defined by the reference inclination as input and actual inclination as output. The block diagram shown in Figure 13, derived from the diagram shown in Figure 3, describes the new plant behavior. Note that internal motor variables $\vec{\theta }$ and $\vec{\omega }$ are vectors, because each motor has its own position control loop. The neck kinematics block converts the individual motor positions to orientation and inclination angles, and the inclination sensor will then convert these angles into plant output signals that will be used in the new feedback loop.

Due to its characteristics, the plant can be modeled as first order system, considering that motion is slow; that is, a low crossover frequency must be used as specification. For instance, using *w*_*cg*_ = 1*rad*/*s*, a standard neck movement is granted while keeping the motion slow enough to use a first order model.

It is expected that the system parameters depend on the target inclination and tip payload. A series of experiments were done in order to determine what plant parameters change and how they do. System parameters were identified for different inclinations and payloads using a Recursive Least Squares (RLS) method.

Several identification experiments were performed attaching different masses at the tip, while commanding an inclination increasing trajectory (input 0−10*deg* in 10 s). During the experiment, the system was continuously identified within each time fraction (sampling time *Ts* = 0.025 s). Both time constant(τ) and gain (*k*_*n*_) of the system were captured and stored in an array for plotting.

**Figure 14:**
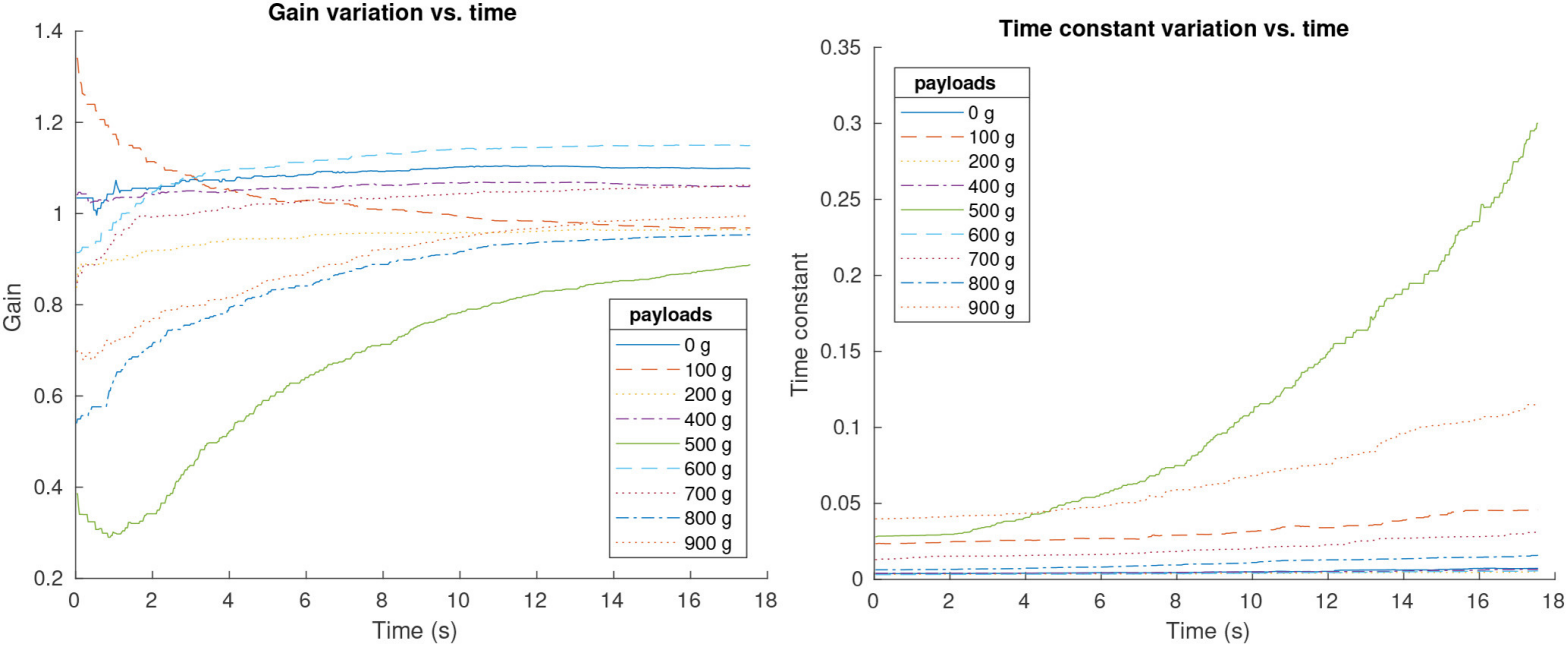
System identification results. System gain variation over time **(Left)** and time constant variation over time **(Right)**.

The results in Figure 14 show that both disturbances impact the system parameters in different ways. As expected, gains converge to values close to one as time grows, and different payloads make a difference in the final value. Time constants do not converge but grow together with the inclination, as expected, since the more the spring bends, the more the motor torque loads increase. Again, payload plays an important role in the time constant curves.

It is important to note that while parameter variation appears to follow a trend in the case of inclination variations, it is not the same for payload changes. As a growing inclination always increases the motor load, different payloads can affect the motors in different ways. Some payloads will help bending in some positions and prevent it in others.

### 7.1. Plant Parameters

Orientation variable accuracy is enough using an open loop configuration, as shown in section 6. As the feedback loop will only include inclination error, an inclination only model can be used. Previous experimental results show that a first order plant like the one defined by Equation (16) can be used, but also shows a substantial parameter variation depending on payload and inclination. The plant will be modeled as a Single Input Single Output (SISO) system with inclination input (*deg*) and inclination output (*deg*).

(16) $$\begin{array}{l} G\left(s\right)=\frac{k_{n}}{\tau s+1} \end{array}$$

The average values of gain and time constant will be used for the system model, and a robust controller will be applied in order to guarantee a robust performance. The average system parameters chosen are the mean values for the experiment with a payload of 500 g shown in Figure 14. For this case, *k*_*n*_ = 0.65 and τ = 0.156, resulting in the following first order system:

(17) $$\begin{array}{l} G\left(s\right)=\frac{0.65}{0.156s+1} \end{array}$$

### 7.2. Fractional Order Control Feedback

Once the system is defined, the control scheme can be designed in order to fulfill the new control specifications. First of all, the aim of the loop is to improve accuracy. Since a zero steady-state error is needed, a fractional integral controller will be used. Second, motion must be slow; therefore, the crossover frequency must be small (*w*_*cg*_ = 1*rad*/*s*). And third, in order to ensure system stability, a large phase margin will be used (ϕ_*m*_ = 100). As a summary, the new control specifications are as follows:

- *E*_∞_ = 0
- *w*_*cg*_ = 1*rad*/*s*
- ϕ_*m*_ = 100

**Figure 15:**
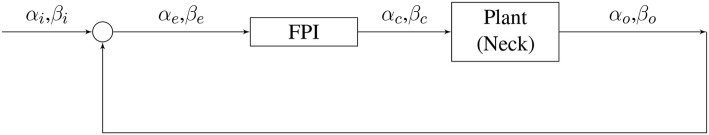
Fractional order control feedback for the neck system described in Equation (**??**).

The block diagram of the proposed control strategy is shown in Figure 15.

**Figure 16:**
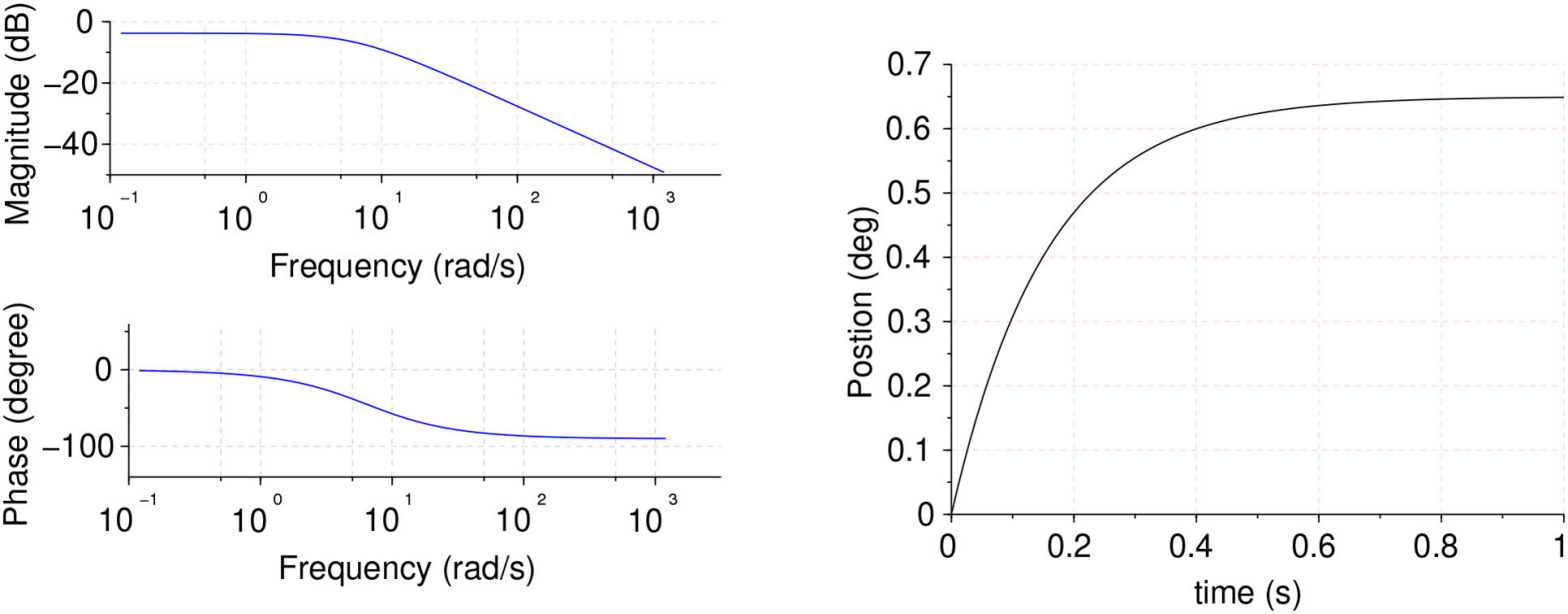
Plant model frequency response **(Left)** and time unitary feedback response **(Right)**.

Plant model frequency and time responses are shown in Figure 16.

Using the plant model shown in Equation (17), a fractional integral control was implemented using Monje's method (Monje et al., 2008b), seeking also flat phase slope robustness specification in order to deal with the discussed plant uncertainties. The parameters obtained were *kp*:0.2702503; *ki*:1.4920678; *ei*:−0.9, resulting in the following controller transfer function:

(18) $$\begin{array}{l} C\left(s\right)=0.2702503+1.4920678s^{-0.9}, \end{array}$$

**Figure 17:**
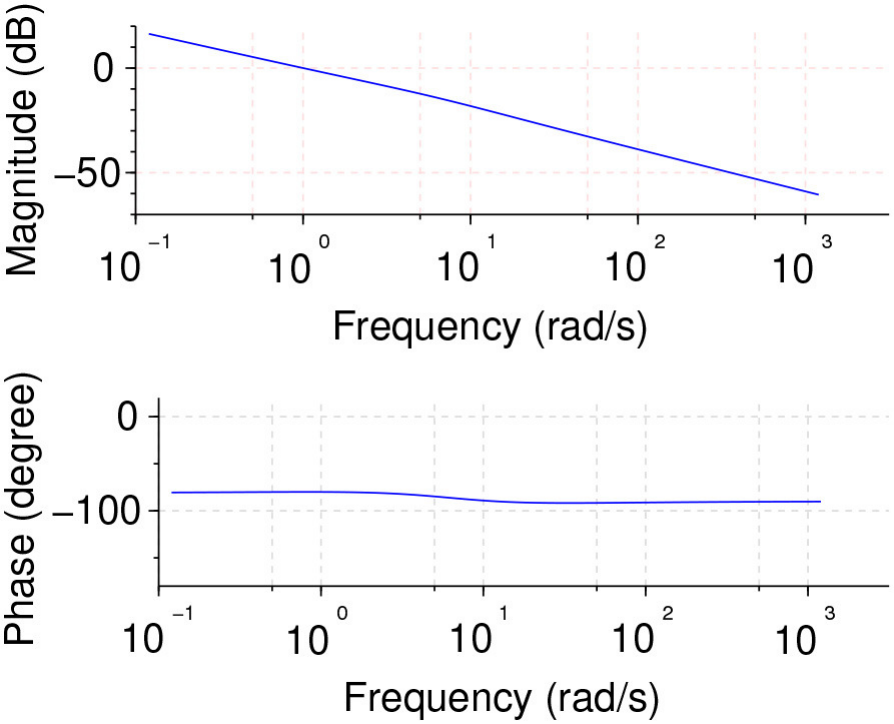
Open loop Bode diagram for neck plant and controller showing the flat phase slope specification.

The Bode diagram for the system and controller in open loop configuration is shown in Figure 17, where it can be seen that the control specifications are met.

### 7.3. IMU-Based Control Experiments

**Figure 18:**
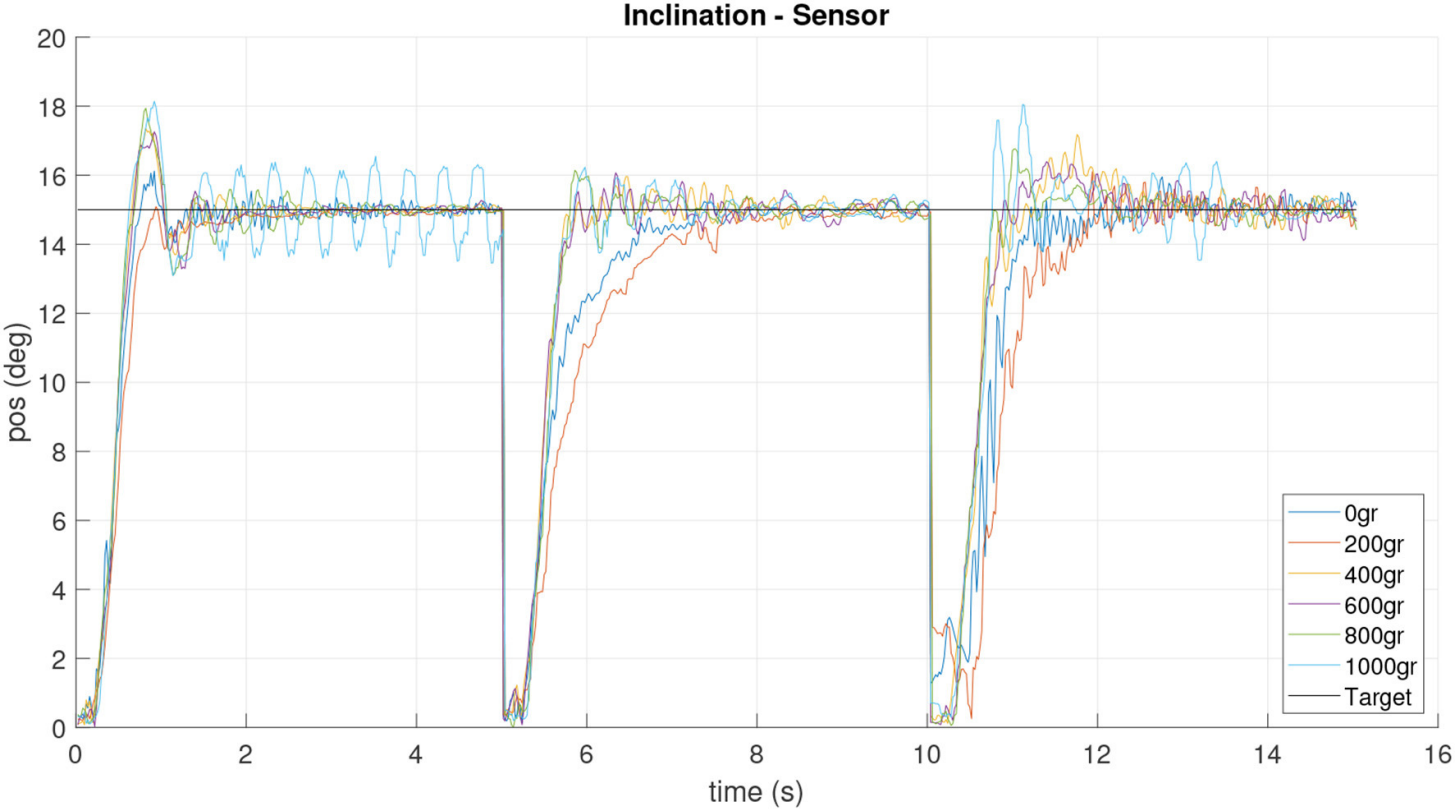
Sensor inclination measurements during the closed loop operation.

**Figure 19:**
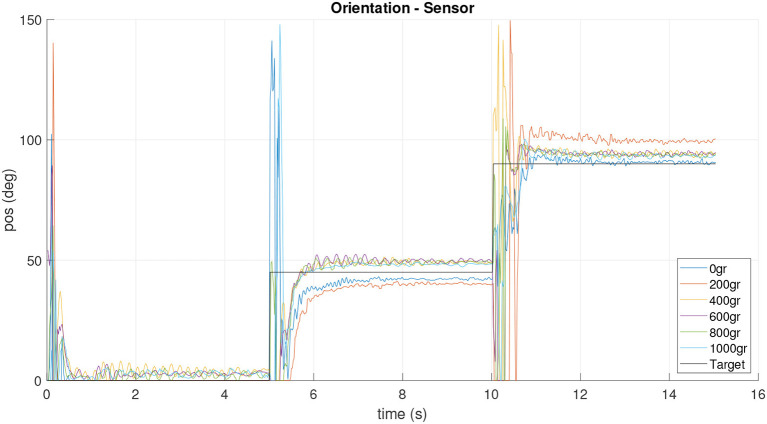
Sensor orientation measurements during the closed loop operation.

After controller design and implementation, the feedback loop was tested in the real platform by means of orientation and inclination experiments. The neck targets were set to three different points in the work space: three steps with 15*deg* inclination and 0, 45, and 90(*deg*) orientation while the IMU sensor was used to both capture the actual neck tip position and for feedback loop.The tests can be visualized in the video at https://bit.ly/2MAxF2J. The results are shown in Figures 18, 19, where a zero error in steady state is shown for inclination, and as expected, orientation shows a good accuracy, even controlled without a feedback.

Robustness is clearly achieved for the payload values surrounding the chosen default system (500 g payload), at sight of the performances for the 400 and 600 g payloads. These are similar both in overshoot (10% max, 6.6% min) and settling time (2.2 max, 1.4 min). Wider mass variations show a further performance change, mostly in the overshoot, and finally, for masses close to the maximum payload (1, 000 g), the behavior approaches to instability.

**Figure 20:**
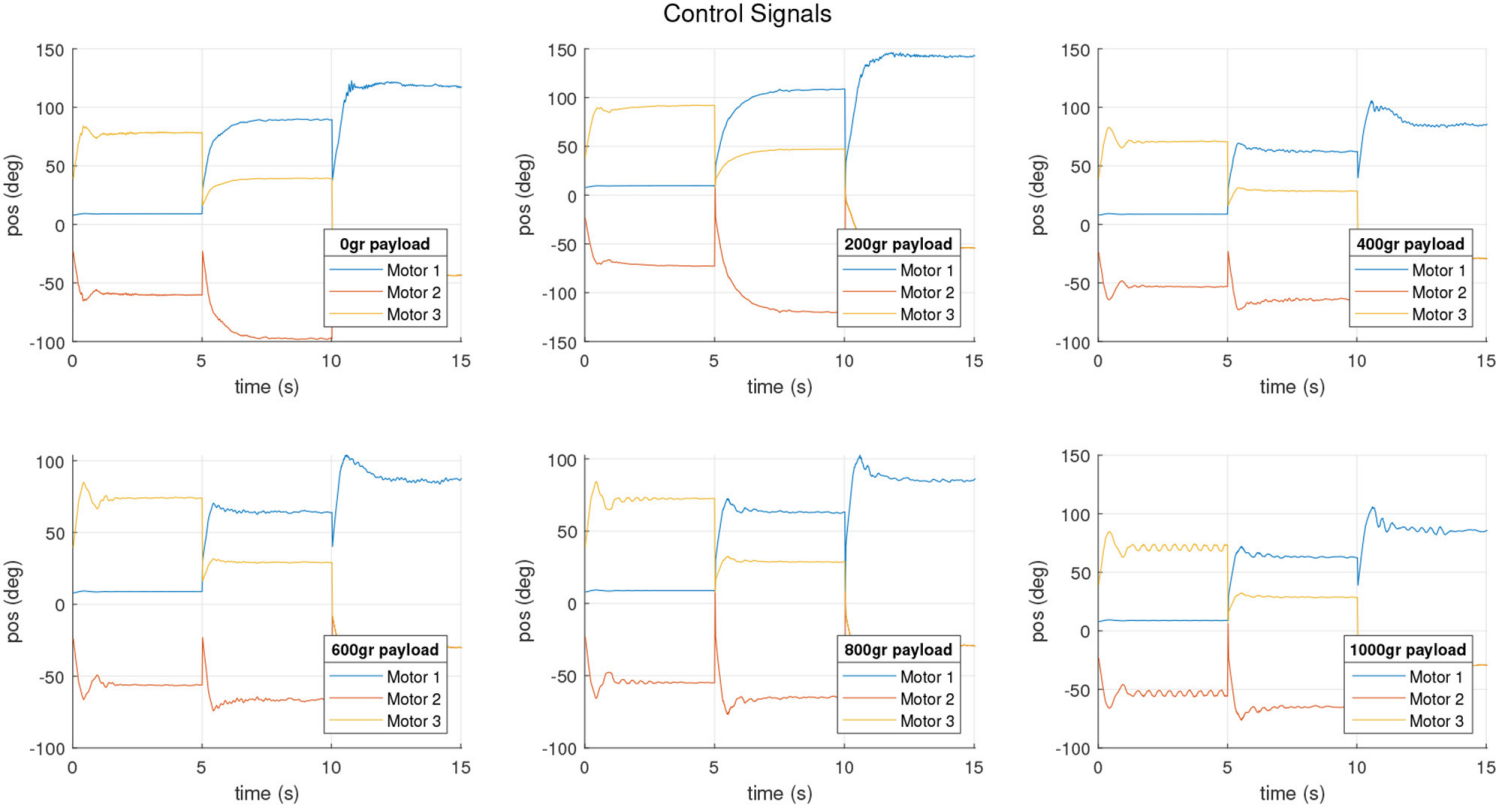
Control signals obtained during the closed loop operation.

Control signals for that experiments are shown in Figure 20. No saturation or errors were detected in the course of the experiments.

**Figure 21:**
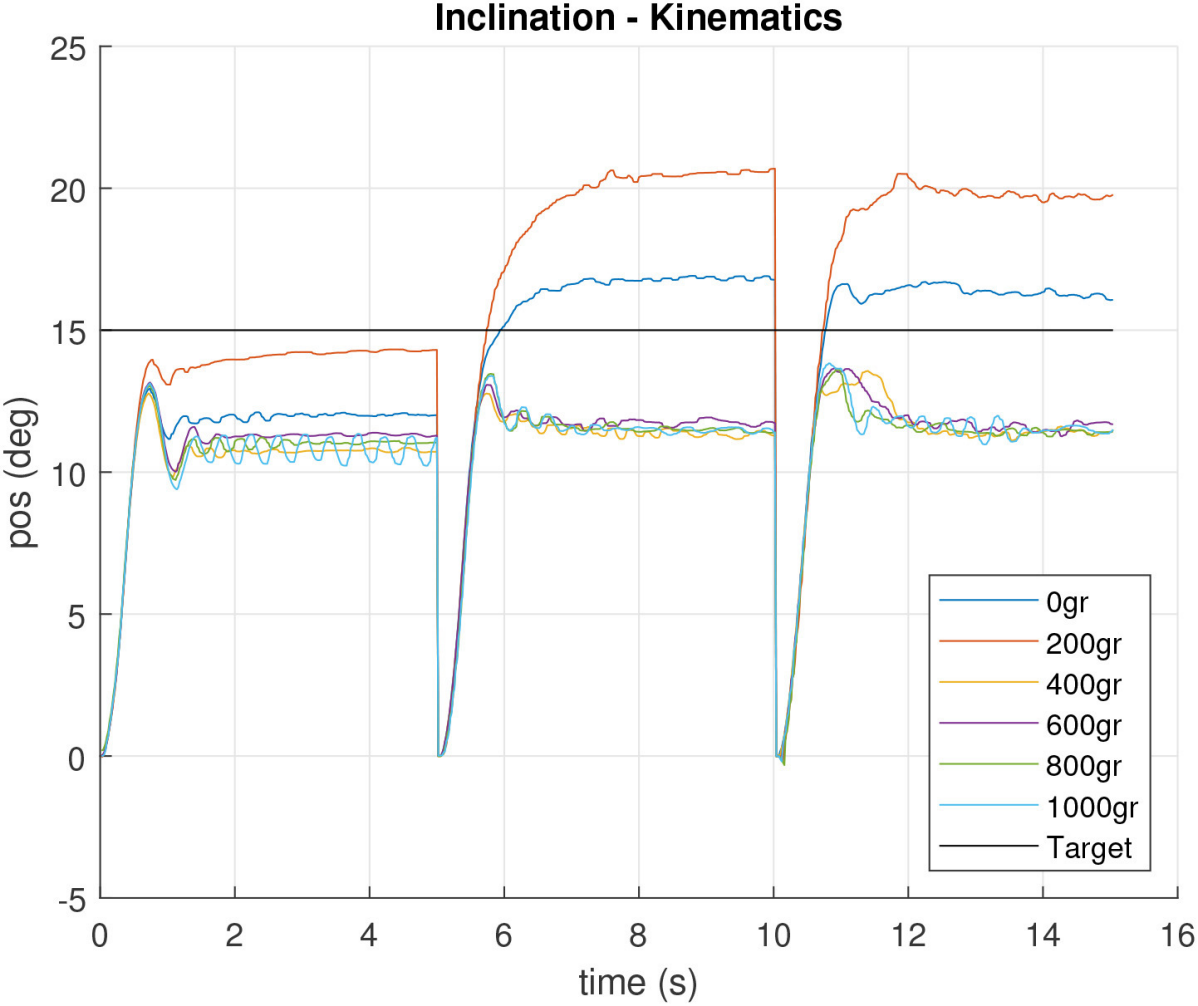
Theoretical inclination according direct kinematics for actual inclination feedback controlled motor positions.

**Figure 22:**
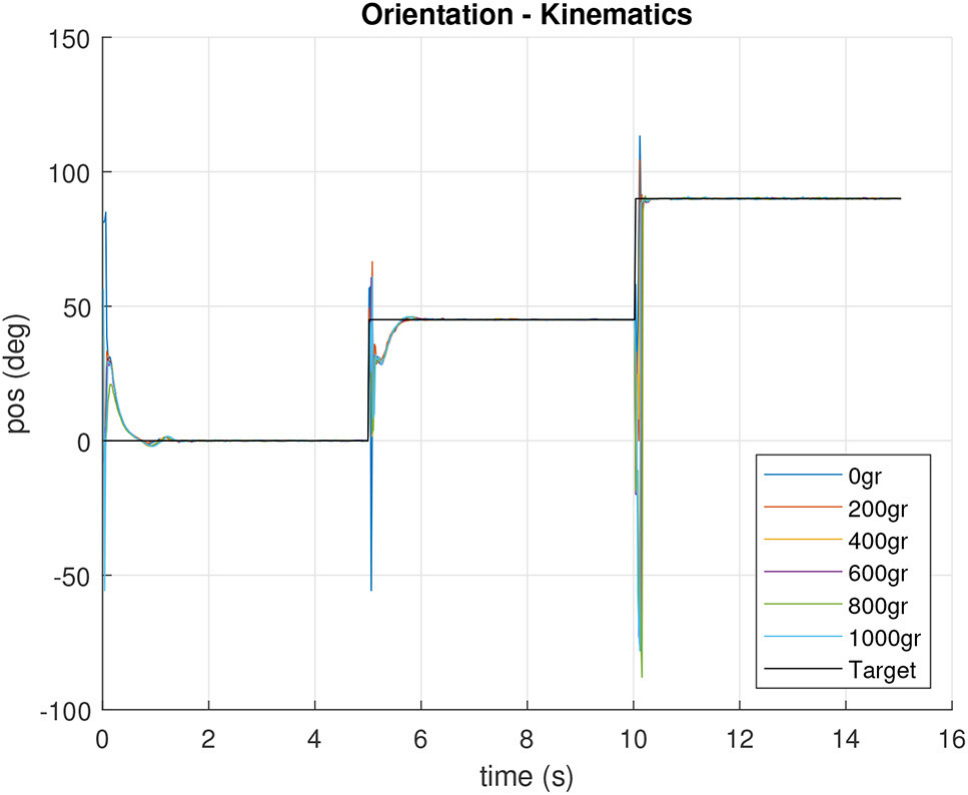
Theoretical orientation according direct kinematics for actual inclination feedback controlled motor positions.

In order to show the differences between the first and the second control approaches, Figures 21, 22 shows the inclination and orientation results according to actual motor positions and direct neck kinematics. It can be observed how big the error can be in some situations, like in the cases of 0 and 200 g. It is evidenced that low payloads make it harder for the system to reach a target inclination.

Normally, the higher the inclination, the harder to bend the spring and the higher the inclination error. In fact, according to Figure 21, the inclination mismatch detected in previous sections was not due to the mass load, but to the lack of mass. Once the mass is bigger, the correction is needed in the opposite direction in order to hold the right target inclination.

## 8. Conclusions

This paper has presented a soft robotic neck with two DOF providing pitch and roll movements. We have solved the kinematics problem considering the dimensions and mechanical properties of the spring.

The tests carried out allowed us to verify the performance of the platform and validate the kinematics through encoders data.

Knowing that plant parameters change with inclination and payload (and so do motor parameters), a robust controller was used in order to fulfill specifications despite system variations. The fractional order robust controller grants that motors reach and hold position targets established by the kinematics while showing similar performances regardless inclination or load variations on the neck. The neck platform was tested with loads up to 1 kg, presenting a robust mechanical and control performance.

Additionally, an IMU sensor MPU92-50 was implemented to better characterize the real behavior of the neck platform. From the measurements obtained, we concluded that an error is obtained when comparing the neck position estimated through the kinematic model and the real one measured by the IMU sensor.

As a novel contribution of the work, an IMU-based control loop has been closed, using another fractional order controller. The experimental results show that the system performance is now more accurate and robust to load variations.

Right now, the prototype is a test bench and all tests were done on a flat surface, without mounting the neck on the humanoid. In the future, we will integrate the neck into our humanoid robot, which has an IMU sensor between the torso and the waist. This sensor will be used as a reference.

Besides, as a further research step, a new soft material link will replace the spring in order to improve the performance of the platform.

## Data Availability Statement

The datasets generated for this study are available on request to the corresponding author.

## Author Contributions

All authors listed have made a substantial, direct and intellectual contribution to the work, and approved it for publication.

### Conflict of Interest

The authors declare that the research was conducted in the absence of any commercial or financial relationships that could be construed as a potential conflict of interest. The handling Editor declared a shared affiliation, though no other collaboration, with the authors LM, CM, LN, JM, and CB.
